# A Victims’ Coping Strategies Model of School Bullying Coping: A Grounded Theory Study of Chinese Students’ Retrospective View

**DOI:** 10.3390/bs16040481

**Published:** 2026-03-24

**Authors:** Jiaying Wang, Qianqian Zhang, Tiantian Yu, Zhongping Zhao, Zhanhong Zhu, Jielei Jiang

**Affiliations:** 1 College of Education and Psychology, Shaoxing University, Shaoxing 312000, China; 2 Zhejiang Shaoxing Xinghua Qianjin Digital Technology Co., Ltd., Shaoxing 312300, China; 3 Shaoxing Basic Education Collaborative Innovation Research Center, Shaoxing University, Shaoxing 312000, China

**Keywords:** school bullying, coping strategies, victims’ coping strategies model in China, developmental and cultural perspective

## Abstract

Coping serves as a protective function in students’ responses to school bullying. Previous studies have proposed several models to explain how victims cope with school bullying, but most of these frameworks were developed in Western contexts. Grounded in these frameworks, this qualitative study explores how victims cope with different developmental stages of school bullying within the Chinese cultural context. Using grounded theory and constant comparative analysis, we analyzed retrospective self-reports from 67 Chinese university students who described bullying experiences from elementary to high school. The analysis identified four key coping categories: emotional response, endurance and avoidance, cognitive reconstruction, and action-oriented resistance. Based on these coping strategies, we developed a Victims’ Coping Strategies Model structured along two axes: engagement–disengagement and a cognitive–emotional to cognitive–behavioral continuum. By capturing the complex interplay of internal and external strategies influenced by Chinese sociocultural norms, the model demonstrates the developmental and context-dependent nature beyond static classifications of coping strategies. The findings contribute to cultural and developmental understandings of victim responses and inform practical implications for intervention.

## 1. Introduction

Coping is the victim’s response to potential stressors through personal regulation or efforts to manage specific external or internal demands that are appraised as exceeding their resources (Compas et al., 2001; Lazarus & Folkman, 1984). In the school bullying context, which is repeated, intentional aggression in relationships marked by a power imbalance, victims’ coping entails strategies to manage victimization-related stress (Hunter & Boyle, 2004; Olweus, 1993).

Research indicates that many of the victims tend to adopt coping strategies when confronted with school bullying (Skrzypiec et al., 2011). From the resilience perspective, effective coping can serve as a protective factor, potentially mitigating the adverse psychological and academic consequences of victimization (Baldry & Farrington, 2005; Hemphill et al., 2014). While general coping models provide a foundational understanding, school bullying coping is increasingly viewed as a dynamic, context-dependent process rather than a static trait, necessitating examination within specific sociocultural and developmental frameworks to deepen understanding and inform effective interventions (Iordache et al., 2025; Njelesani et al., 2025).

### 1.1. General Coping Models and Their Application to School Bullying

Some well-established theoretical models have been employed to conceptualize how individuals cope with school bullying. Roth and Cohen (1986) proposed an approach–avoidance model, where approach involves confrontation and avoidance involves withdrawal. The seminal model by Lazarus and Folkman (1984) distinguishes between problem-focused coping (aimed at altering the stressful situation) and emotion-focused coping (aimed at regulating emotional distress). Subsequent adaptations have expanded this binary conceptualization of coping. For instance, Davis et al. (2014) incorporated cognitive mechanisms related to victims’ internalized appraisals of bullying, while others have emphasized behavioral dimensions, categorizing strategies as active (outwardly expressed) versus passive (involving acceptance or negative self-targeting) (Kokkinos et al., 2014). The control-based model differentiates primary control (attempts to change the external environment) from secondary control (efforts to adapt oneself to the situation) (Weisz et al., 1995). Similarly, the engagement–disengagement framework contrasts engagement coping (directly addressing the stressor) with disengagement coping (mentally or behaviorally distancing from it) (Compas et al., 2001; Sontag & Graber, 2010).

Despite this theoretical richness, a significant gap exists between these generalized frameworks and the nuanced reality of coping in bullying contexts. Empirical studies catalog a wide array of strategies reported by victims, including seeking social support, using avoidance or ignoring, employing cognitive reappraisal, internalizing (e.g., self-blame, rumination), externalizing (e.g., aggression, retaliation), and using humor or submission (Garandeau et al., 2023; Garnefski & Kraaij, 2014; Skrzypiec et al., 2011; Xie et al., 2022). A key limitation of existing research is the reliance on predefined categories derived from general stress models or prior bullying studies, which may not fully capture the spontaneous and situated nature of victims’ responses, potentially overlooking multiple strategies used in combination or culturally-specific manifestations. A key gap is the absence of a model that integrates diverse coping strategies in a context-sensitive way, systematically organizing the diverse coping strategies that are particularly important in the context of school bullying.

### 1.2. Determinants of Coping Strategy Adoption

The transactional coping theory emphasizes personal and situational variables influencing individuals’ coping strategy adoption when facing school bullying (Hunter & Boyle, 2004). Students’ cognitive and social development progresses with age, enabling them to evaluate potential outcomes and select context-appropriate coping strategies in school bullying (Nixon et al., 2019). Elementary students tend to ignore or warn bullies, seek adult help, and exhibit socioemotional responses. As children transition to adolescence, they increasingly rely on approach strategies like seeking support and less on avoidance strategies (Houbre et al., 2010). Flanagan et al. (2012) found forgiveness effective for grades six to eight students. Eslea (2001) investigated coping strategies among 11- to 15-year-olds, identifying fighting back, doing nothing, and ignoring as the most common, though students employed different sequences across contexts. These results align with studies indicating that younger students prefer constructive strategies (problem-solving, support-seeking, self-soothing), whereas some adolescents disengage from coping (Hunter & Borg, 2006; Parris et al., 2017).

The sociocultural context influences how students cope with school bullying across countries. Studies from Australia indicated that coping strategies employed by students included retaliation, avoidance, ignoring, emotional comfort, problem-solving, nonchalant distancing, externalization, internalization, and help-seeking (from adults/peers) (Skrzypiec et al., 2011). In the United States, students most commonly reported using resilience, cognitive distancing, externalization, self-blame, and social support seeking, whereas students from Thailand tended to rely more on self-blame and tolerance (Grothaus, 2023; Parris et al., 2017; Tenenbaum et al., 2011). Comparative research revealed that help-seeking is a widely accepted coping strategy among British students, while Japanese students are more likely to ask the bully to stop directly, tolerate the bullying, or alter their own behavior (Kanetsuna et al., 2006). Reports from European countries, such as Italy, showed that students utilized diverse coping strategies, including support seeking, arguing with bullies, nonchalance, and distancing (Menabò et al., 2025). In China, the most commonly identified coping strategies were help-seeking, humor, avoidance, defense, and cognitive reappraisal (Huang & Wan, 2025; Xie et al., 2022; Zheng et al., 2023; Zhou et al., 2023). Although coping strategies among victims from varied backgrounds are highly diverse, they sometimes share common core elements across contexts.

### 1.3. Present Study

Although previous studies have identified diverse bullying coping strategies and their key influencing factors, it still remains limited in two respects. First, existing models, largely adapted from general stress frameworks, may inadequately reflect the unique coping strategy patterns that emerge in real bullying contexts. Second, there is a paucity of integrative models that are simultaneously informed by developmental stages and culture, especially those outside the Western context, such as China.

To address these gaps, this study proposes the Victims’ Coping Strategies Model, aiming to construct a holistic, integrative framework of coping strategies from the retrospective narratives of college students in China. The model is explicitly designed to be developmentally informed, in that it captures coping strategies across elementary, middle, and high school stages, and culturally situated, in that it reflects the Chinese sociocultural context (Hunter & Borg, 2006). In addition, the strategies identified in this study are also mapped onto established theoretical dimensions (e.g., approach–avoidance and engagement–disengagement), connecting the model with existing coping theories.

By taking the qualitative approach, this study could provide richer contextual detail, thereby moving beyond previous quantitative research on college students’ school bullying coping strategies, which was constrained by limited coping categories from survey scales (Hunter et al., 2004; Newman et al., 2010). In addition, integrating multiple developmental stages with consideration of cultural factors contributes to a more context-sensitive and holistic understanding of coping with school bullying (Smith & Caron, 2022).

## 2. Methods

This study employed a constructivist grounded theory design, which allows theories to emerge and be co-constructed from participants’ narratives, with researchers taking an interpretive role (Charmaz, 2006). The inductive nature of constructivist grounded theory supports the development of a model based on victims’ coping strategies from their retrospective written self-reports. In November 2023, by teaching the course Introduction to Education to first- through fourth-year undergraduates, the researchers assigned students a self-written report on their “experiences with school bullying” following a seminar discussion on topical issues such as school bullying. General guidelines for the report were provided, covering aspects that included event recall, psychological state, coping strategies, and subsequent impact. The Institutional Review Board of Shaoxing University, Zhejiang Province (approval no. 2023112602) approved this study. Written informed consent was received from all participants.

### 2.1. Participants

The participants included 80 undergraduate students (48 female, 32 male; M_age = 20.44 years, SD = 1.52) from S University, Zhejiang Province, who reported prior experiences of school bullying. The decision to focus on university students for this study on school bullying was based on three considerations. First, university students generally possess stronger written expression skills, enabling them to provide more complete and detailed accounts of bullying incidents they personally experienced or witnessed during their primary and secondary school years. Second, in retrospect, university students are generally able to recall these experiences more objectively and explain their own state at the time. Third, the practical considerations of convenient sampling. Students were recruited through convenience sampling across diverse undergraduate programs, including freshmen (n = 21), sophomores (n = 18), juniors (n = 23), and seniors (n = 18). Based on Salmivalli’s (2014) role classifications in school bullying, the researchers categorized the self-reports into four types: bully-only (n = 4), bystander-only (n = 9), victim-only (n = 27), and multiple-role (bully/victim/bystander; n = 40). Reports were retained for analysis (n = 67) if they contained the complete victimization narrative or at least 50% of the content describing the victim experience. Among these, 27 were classified as pure victim narratives, and 40 as multiple-role narratives. In keeping with the theoretical sampling rules in constructivist grounded theory, this study focused on the victims’ experience rather than the mixed role identities (Charmaz, 2006). From the multiple-role self-reports, the researchers carefully extracted only the excerpts that described participants’ victim role experiences. To ensure that victim-related experiences were identified consistently, two researchers compared coping accounts from victim-only and multiple-role reports prior to coding and again after the development of the coding framework. This comparison was conducted to assess whether role plurality meaningfully influenced the coping narratives. Through discussion and consensus, no systematic differences were observed in the coping strategies between the single victims and multiple positioning in these school bullying reports.

### 2.2. Procedure

Participants independently completed a written self-report on their experiences with school bullying during elementary, middle, or high school. To ensure standardization and comparability across reports, the researchers provided a structured framework with guiding prompts, which asked participants to recall and describe the bullying incidents, their emotional responses, coping strategies, and subsequent impacts or outcomes, either in elementary, middle, or high school. Within this framework, participants were encouraged to elaborate on their narratives by providing detailed descriptions of their school bullying experiences across the elementary, middle, and high school periods.

### 2.3. Data Analysis

Before data coding and analysis, four researchers, all native Chinese speakers who were familiar with the sociocultural context of school bullying in China, received training and practice in NVivo-based coding and analysis. Based on an initial comprehensive review of the self-reports, the research team developed specific coding guidelines and analytical procedures. Each researcher independently coded approximately 16–17 self-reports. Researchers exchanged their coded reports for cross-checking at each stage of coding. Discrepancies and potential biases were addressed through discussion and iterative review of the raw data until a consensus was reached among the research team. Given the retrospective nature of the data, participants’ recollections may be subject to recall bias or memory reconstruction of school bullying experiences. To enhance the reliability of the analysis, iterative review and researcher triangulation were applied throughout the coding process, with multiple researchers independently reviewing and coding the data.

The data were analyzed using constructivist grounded theory and the constant comparative method (Charmaz, 2006). The researchers imported 67 self-reports into NVivo and iteratively analyzed them in several stages:

(a) Initial coding was performed to identify, label, and extract key concepts from the data. Through coding line-by-line, 43 preliminary nodes were generated, covering bullying contexts (e.g., location, perpetrator identity, frequency, and outcomes) and victims’ coping strategies (Table 1).
(b) During focused coding, the frequent codes were compared and refined. Related codes were grouped into broader categories, and irrelevant nodes were removed. The bullying context codes were used to situate, interpret, and support the description and understand the victims’ coping process.
(c) With ongoing memo writing and comparison between new data and existing codes, the categories were further refined. Theoretical saturation was met when no new concepts related to victims’ coping strategies emerged. Four domains were identified.

Because the narrative data were originally written in Chinese and later translated into English for reporting, two bilingual researchers (Chinese-English) cross-checked the translations. When discrepancies arose regarding whether the translations fully preserved the original meanings and captured the cultural nuances, a third bilingual expert (Chinese-English) was consulted to reach a consensus.

## 3. Results

Although the conceptual interpretation is prioritized by constructivist grounded theory, descriptive indicators could be presented as the relative salience of coping responses in the narrative self-reports (Charmaz, 2006; Miles et al., 2014). To better demonstrate the relative prominence of different coping strategies, we counted the narrative segments from students’ self-reports, without implying frequency of prevalence among participants.

Based on the patterns of school bullying coping strategies identified in participants’ self-reports, Table 2 presents the domains, categories, segments reported, and rank orders. Among the 67 self-reports, 25 described bullying experiences in elementary school, 22 in middle school, and 20 in high school. From the participant self-reports, emotional response strategies were most commonly observed (66 segments), followed by action-oriented resistance strategies (47 segments). Endurance and avoidance strategies (37 segments) and cognitive reconstructuring strategies (29 segments) appeared less frequently in the coded data. At the category level, emotional involution (48 segments) and leveraging strength for resistance (33 segments) were the most salient coping strategies. Patterns in students’ coping strategies across developmental stages are presented in Table 3.

The emotional response strategies were more commonly described in elementary school and gradually declined in later school periods. In particular, emotional release was most visible in elementary school narratives and became much less common in middle and high school self-reports. Emotional involution appeared relatively stable across stages. Endurance and avoidance strategies tended to decline from elementary school to middle school, followed by a slight increase in high school self-reports. Within this domain, bullying acquiescence appeared more prominently in higher school stages, whereas bullying fantasization was primarily in elementary school self-reports and rarely in high school narratives.

Cognitive reconstruction strategies were most commonly observed in middle school narratives. The segments of rationalization for bullying continuously increased from elementary school to high school. Negative self-perception was relatively similar in elementary and middle school, but it appeared less in high school narratives. Action-oriented resistance strategies were reported across all developmental stages. Within this domain, leveraging support from friends was less represented in elementary school narratives, was not observed in middle school, and appeared more in high school narratives.

### 3.1. Emotional Response Strategy

The emotional response is a coping strategy employed by school bullying victims to manage both immediate affective states (e.g., sadness, loneliness) and sustained emotional changes, including stress-induced responses. Emotional release and emotional involution are two categories in this domain.

#### 3.1.1. Emotional Release

In coping with school bullying, victims engaged in emotional release through crying (e.g., sobbing, wailing, whimpering, blubbering) and temper outbursts. Immediate emotional release occurred at the time of bullying incidents in elementary school, whereas in middle and high school, such responses tended to be prolonged or delayed. Notably, many victims employed covert emotional release as a self-regulatory strategy to conceal perceived weakness or vulnerability. For example, an elementary school victim reported: “I truanted and hid in the restroom crying heartbreakingly” (Student 37). A middle school victim described: “I was angry with myself about being laughed at for my appearance. I cried secretly late at night, which caused dacryoadenitis and photophobia” (Student 60). However, such “hidden emotional release” was generally ineffective and occasionally exacerbated bullying. As Student 28 recounted after property embezzlement: “I went back to the dormitory, hid under the blanket, and sobbed”, yet the bullying persisted: “The next day, they requested more money.”

#### 3.1.2. Emotional Involution

Drawing on the sociological concept of involution (Geertz, 1963), we use the term “emotional involution” to interpret participants’ descriptions of inward-turning, difficult-to-regulate negative emotions arising from school bullying. Victims used terms such as “scared all the time” (Student 31), “grievance, and despair… until now” (Student 4) to describe the negative emotional state internalized long-term that profoundly impacted their interpersonal relationships and development. One middle school bullying reported by Student 18: “I was very depressed when I was ostracized, which made me indifferent to everyone and everything.” Student 6 reported the school bullying in high school: “I felt I was on the verge of a breakdown and suicide if someone trigger my bullying memory. Once upon the scenes of being bullied replay in my mind, I still have such feelings.” The stagnated and involuted emotions lead to the victim’s physical and social disorder, such as “stomachaches, nightmares, and insomnia” (Student 43) and “worried about initiating new social relationships” (Student 29).

### 3.2. Endurance and Avoidance Strategy

Victims cope by retreating, submitting, or evading to endure and avoid bullies or bullying situations. The endurance and avoidance strategies include bullying acquiescence, bullying avoidance, and bullying fantasizing.

#### 3.2.1. Bullying Acquiescence

Acquiescence reflects victims’ conciliatory attitudes toward school bullying by remaining silent and enduring in the hope of terminating the bullying and achieving a sense of peace. The acquiescence strategy adopted by the victims may be influenced by values of self-restraint to attain harmony. For example, Student 56 reported:

As my classmate continued to extort money, I didn’t even ask him when he would return the money and I only wanted to get some money to give him. When I got home, I found ten yuan in the closet of my parent’s room. I took it out when my grandfather didn’t notice and gave it to the bully the next day to not make the trouble.

In fact, many victims recognized that acquiescing to bullying was ineffective in reducing or ending the abuse, even though they continued to use this coping strategy. The outcome of such “appeasement” was not the bullies’ sympathy or mercy, but rather intensified bullying. In one middle school physical bullying, a student’s leg was forcefully stabbed with a pencil by a bully, but the victim’s acquiescence escalated it:

I was afraid that resisting would draw more attention, so I tolerated the pain each time, hoping the bullying would stop. However, my inferiority and timid personality made the bullying only worsened. He changed the blunt end to a sharp point and viciously stabbing it into my leg. Until one day in class, he took out all of his pencils and simultaneously stabbed my leg with the pencils’ sharpened points.

(Student 9)

#### 3.2.2. Bullying Avoidance

Some victims make efforts to evade and distance themselves from the bullies to avoid further school bullying. As an active coping approach, avoidance aims to reduce or stop bullying and can help protect victims from additional harm in the short term. Student 20 reported:

Since then, whenever I went to school, the first thing I did was check if the bully was there. If he was there, I chose to avoid him and went elsewhere. These actions are instinctive. I don’t know if others who have experienced school bullying have the same response afterward. At that time, I couldn’t understand my behavior, but I always searched for the bully’s presence wherever I went. If he was present, I maintained a safe distance.

#### 3.2.3. Bullying Fantasizing

When individuals feel unable to cope with the distress and pressure from the school bullying, they may temporarily escape the troubling circumstances through fantasizing to achieve inner balance and obtain the satisfaction that may not be attainable in real life. Elementary school victims reported fantasies such as “turning back time to before the bullying occurred” (Student 67), “removing some characteristics that cause being targeted” (Student 1), or even “imagining bullies and witnesses suddenly forgetting the bullying immediately” (Student 46). By harnessing this “supernatural power”, victims achieve temporary self-comfort. Student 55 described: “I look at myself in the mirror, imagining I will be beautiful and telling myself that the bully would disappear when I wake up the next day.”

### 3.3. Cognitive Reconstruction Strategy

In cases of school bullying, victims may engage in cognitive reconstruction to develop new interpretations of the bullying experience, themselves, and their social environment. This strategy comprises two dimensions: rationalization of bullying and negative self-perception.

#### 3.3.1. Rationalization for Bullying

When conflicts or pressures from school bullying become intolerable, victims may reduce the perceived threat and emotional impact through cognitive distortion. This involves misinterpreting facts, others’ intentions, or emotional cues to create “logical” justifications that mask pain and achieve cognitive equilibrium. One middle school student rationalized school bullying to seek cognitive balance: “Humans are all selfish and flawed. The school bullying on me was just a reflection of human selfishness and weaknesses.” (Student 61) Through such rationalizations, victims attain internal cognitive reconciliation, releasing negative thoughts. Student 39 noted: “I felt that the boy who bullied me was quite unfortunate. He did not perform well in the school and his parents were divorced. Perhaps he envied me. When I think of that, I feel better”.

#### 3.3.2. Negative Self-Perception

Negative self-perception operates as an inherently passive strategy in school bullying. When labeled with derogatory terms such as “ugly”, “idiot”, or “stupid”, victims may internalize these insults and subsequently adopt corresponding self-perceptions. Over time, they may subconsciously accept these negative traits as valid, leading to behavioral and value-based changes. Student 21 reported: “I began to think I was very unattractive, covering my mouth when laughing, and cared about my appearance consciously to prevent being teased.” Student 44 pointed out: “For a long time, I felt I was engraved with an ugly scar to keep me from accepting and liking myself. I trusted that I did something wrong in this world so that I deserve to be bullied.”

### 3.4. Action-Oriented Resistance Strategy

In school bullying, victims take coping actions to resist and address the bullying, either independently or through external support-seeking.

#### 3.4.1. Individual Resistance

To confront or resist school bullying, victims may rely on their own strength. They try to change others or change themselves to eliminate or end the school bullying.

##### Resistance to Changing Others

Physical and verbal resistance strategies represent two primary approaches to modify others’ behavior in school bullying. Elementary and middle school students tend to use more physical resistance, but this strategy may not lead to positive outcomes in school bullying:

He came to ask me for money again, but I had none that day. Unable to endure further, I believed displaying force would deter the bullying. When I struck him, he escalated aggression, kicking me repeatedly. My resistance intensified the bullying.

(Student 49)

High school students prefer using verbal resistance strategies, including direct questioning, counterargument, or dialogue with bullies, bystanders, or other involved parties in school bullying. Student 2 reported a case where her friend initiated and disseminated rumors among classmates, leading to teasing and social avoidance. She confronted the bully and called out the other students who had spread the rumors during an argument:

In their presence, I inquired, “Do you feel that I verbally attacked you or made you uncomfortable? If so, please specify which words were inappropriate.” When she failed to provide clarification, I continued, “Since you cannot specify any words, why did you tell others that I insulted you? You spread rumors across the school to gain sympathy and pity from people who don’t know the truth. If you truly believed I had wronged you, you could have spoken to me directly, and I would have clarified.” After this, she had nothing to say and apologized.

##### Resistance Through Self-Change

Victims try to enhance their personal strength or develop themselves to resist school bullying. Some students improved their academic performance and skills to gain respect from bullies and cover their weaknesses. Student 48, who spread rumors in elementary school, mentioned: “After my parents learned about my interest in music, I studied piano despite family financial pressure, which earned respect and changed the way bullies only focused on my height, to appreciating my talent, and this skill boosted my confidence.” The affirmation of personal abilities changed the bullying that occurred in middle school: “I endeavored to achieve top 10 class rankings. Gradually, those who used to mock me with nicknames no longer dared to do so.” (Student 36) Other students sought new social connections. Student 24 described middle school experiences: “I tried to build a relationship with some students who have some influence in the school. They also treated me as a friend, feeling that the closer I am to them, the safer I am, and no physical or verbal bullying or extortion to me anymore.” Student 57, who was isolated and excluded in her dormitory in high school, reported: “I tried to rely less on my roommates and chosen to make friends with other classmates. Even though we were not in the same dormitory, they gave me a lot of support to get through the toughest period.”

#### 3.4.2. Leveraging Social Support for Resistance

Leveraging social support and assistance from individuals or groups constitutes an effective strategy for victims to resist school bullying. Based on the source of support, such resistance mechanisms may derive from teachers, parents, and peers.

##### Leveraging Social Support from Teachers

The aims and effectiveness of leveraging teacher-provided social support vary across individuals and contexts. Some victims valued teachers’ authority when seeking help: “I think teacher X owns the ‘largest’ power in my class. We should all listen to her” (Student 32). Some victims obtained effective support: “My math teacher intervened immediately to stop the bullying behavior.” (Student 64) However, some bids for help were unsuccessful when educators misclassified incidents as mere pranks or playful behavior rather than bullying. Student 23 reported:

I raised my hand to signal the teacher, but it was the key time in the lecture, and the teacher did not permit me to speak. It wasn’t until we were assigned to group discussions that she noticed my distress. Given that we were still in class, she just said to the student who bullied me, “Did you just bully her? Don’t be mischievous next time.” To the teacher, this was nothing more than a harmless joke among classmates.

Experiences with dismissive responses eroded victim trust: “I didn’t report this time because my experience with teachers’ (help) showed no meaningful change would occur and I will not ask the teachers’ help anymore.”

(Student 41)

Yet when some students consider seeking teacher support they may perceive possible negative consequences of reporting school bullying. One student reported a fear of retaliation: “After being bullied, my first instinct was that telling a teacher felt like gossiping, which might provoke more intense bullying.”

(Student 5)

##### Leveraging Social Support from Parents

Some victims actively seek parental support by disclosing bullying incidents, while others passively rely on parental strength: “No one wants to help me get rid of the bullying, so I place my last hope in my parents.” (Student 3). When effectively leveraged, parental strength can alleviate bullying-related distress, as demonstrated by:

My mom went to school, reported the incident to the teacher, and requested intervention, which finally ended a half-month nightmare. Though some peers still gave sideways glances, they no longer mocked me. My mom cared my feeling and asked me if I would like to transfer to other schools. I answered yes immediately.

Parental support manifests in various forms: “canceling boarding” (Student 12) or “expressing sympathy and providing comfort” (Student 34). Conversely, some parental responses lack constructiveness, including “advising to ignore bullies instead of focusing on studies” (Student 7), “minimizing bullying significance while counseling not to dwell on it” (Student 14), or “blaming the child for perceived weakness” (Student 38).

##### Leveraging Social Support from Friends

Peer support manifests in three distinct forms. Active counterattackers assist victims in confronting or resisting bullies. While this may occasionally halt bullying, more frequently, both victims and their peers experience severe retaliation. Student 58 reported: “My friend intervened during the bullying incident, but later suffered harsher revenge, the bully poured soup over both our faces. On another occasion, when my friend searched for belongings in the dormitory, the bully forced her head into the wardrobe.”

Silent guardians, though unable to stop bullying directly, provide emotional support to victimized peers. Student 19 noted: “I told my friends but couldn’t urge them to fight back. They only offered comfort, without solutions.”

Betrayers act as accomplices to bullies. Victims seeking peer support may instead become targets of betrayal. Student 10 described: “A classmate posed as ‘class leader’, scolding me publicly. Rather than helping, my friends joined the bullies to mock me.”

### 3.5. Victims’ Coping Strategies Model in China

**Figure 1:**
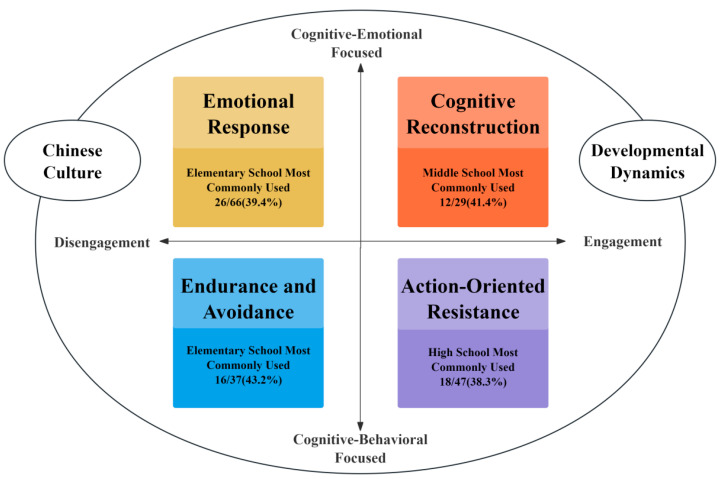
Four-Quadrant Victims’ Coping Strategies Model in China.

A four-quadrant model was developed by drawing on students’ coping strategies, with the quadrants defined by two axes representing tendencies in coping strategy adoption (Figure 1). The axes were determined after a careful review and analysis of the narrative data and the emergent categories in this study, and were then mapped onto existing frameworks that appropriately fit the observed patterns. The horizontal axis represents the proactive dimension of coping responses, grounded in the engagement–disengagement coping framework (Compas et al., 2001; Rudolph et al., 1995; Sontag & Graber, 2010). The vertical axis extends Davis et al.’s (2014) cognitive–behavioral model by integrating emotional factors, forming cognitive–emotional and cognitive–behavioral orientation dimensions. Although these prior models informed the axis selection, the classification of strategies, students’ developmental dynamics, and cultural embedding within the Chinese school bullying context were inductively constructed from participants’ narratives, consistent with the principles of constructivist grounded theory (Charmaz, 2006).

Emotional response represents a disengagement-oriented coping strategy wherein victims respond to school bullying through spontaneous emotional expressions without attempting to resolve the situation. For instance, a victim might report: “I was punched… I went back to my seat and started crying immediately.” (Student 8). When employing this strategy, victims first cognitively recognize the bullying, followed by an emotional reaction, which can be conceptualized as cognitive–emotional coping. Endurance and avoidance are categorized under disengagement-oriented coping; these strategies involve acknowledging the bullying but responding behaviorally after this cognitive realization. For example, a student reported: “I endured and gave him all my money… I deliberately kept my distance whenever I saw him.” (Student 33). In contrast, cognitive reconstruction is a cognitive-engaged approach. Although it involves mental processing, it does not always lead to positive outcomes and may be accompanied by negative emotional components, such as persistent shame despite external reassurance. Action-oriented resistance, on the other hand, exemplifies an engagement-oriented coping strategy, where victims cognitively assess the severity of the bullying and adopt proactive behavioral responses, such as reporting the incident to authorities.

## 4. Discussion

This study presents a holistic view of coping strategies used by Chinese college students reflecting on their past experiences as victims of school bullying from elementary to high school. Four major domains of coping were identified: emotional responses, endurance and avoidance, cognitive reconstruction, and action-oriented resistance. Based on these findings, a model of victims’ coping strategies is proposed.

### 4.1. Patterns and Functions of School Bullying Coping Strategies

Victims most frequently used the emotional response strategy as a central coping mechanism for school bullying. Emotional involution was more frequently reported than emotional release, suggesting that students were more likely to turn negative emotions inward than to express them overtly. Participants’ narratives suggest that being exposed to school bullying may prompt victims to use dysfunctional cognitive emotion regulation, such as catastrophizing school bullying experiences and dwelling on them without seeking solutions to regulate or manage their emotions (Vacca et al., 2023). Unresolved negative emotions may accumulate over time and leave prolonged distressing impacts on the victims’ mental health (Arnarson et al., 2015; Evans et al., 2017). In contrast, emotional release can alleviate some of the stress associated with school bullying. Although shouting or crying represents a disengaged coping strategy, it serves as stress discharge (Syah, 2023). Occasionally, emotional release occurs in a covert manner, such as hiding to cry. Some studies explain that this type of coping allows victims to avoid appearing ashamed and vulnerable while seeking a safe environment in which to express their real emotions (Cowie & Berdondini, 2002; Wójcik & Rzeńca, 2021).

In endurance and avoidance strategies, bullying acquiescence is the most commonly utilized response, followed by fantasizing and avoidance. The predominance of bullying acquiescence indicates that many victims do not actively engage in the coping process, which is consistent with previous studies reporting that victims use the compliance approach for school bullying through self-restraint, concealment, or deliberate ignoring (Imran, 2020). Such responses may reflect victims’ intentions to transfer decision-making authority to others or perceptions of their limited ability to change the school bullying situation (Imran, 2020). Some students used bullying fantasizing, which is similar to wishful thinking and represents a temporary cognitive detachment from the bullying experience. Although this strategy may provide short-term emotional comfort, it does not address the structural root of school bullying (Hunter & Boyle, 2004). Bullying avoidance was not so frequently used in this study, but it has been reported in previous research as a common coping strategy (Houbre et al., 2010; Roth & Cohen, 1986; Skrzypiec et al., 2011; Xie et al., 2022). When students lack the skills to confront school bullying or are not confident enough to actively address it, they may adopt avoidance as a self-protective mechanism (DeVoe, 2007; Han & Zhao, 2024).

Cognitive reconstruction strategies were used least frequently in this study. This strategy involves active cognitive engagement to reappraise and address school bullying, forming a critical component of emotion regulation (Huang & Wan, 2025). However, engaging in cognitive reconstruction can be demanding, which may explain its less frequent use compared to other strategies (Huang & Wan, 2025; Vacca et al., 2023). Moreover, this strategy may not fully mitigate distress or prevent the development of internalized negative self-perceptions, such as repetitive rumination or self-blame (Vacca et al., 2023; Yang et al., 2024). In some instances, victims’ reports suggested that rationalizing bullying incidents served as a way to manage their emotions. Prior research indicates that such reinterpretation can be a means to achieve psychological equilibrium (Xu et al., 2025).

Action-oriented resistance strategies were frequently reported, indicating that many victims cope with school bullying in an engaged and purposeful manner. Rather than responding impulsively, this strategy integrates cognitive appraisal to guide behavioral responses to bullying situations. In this domain, victims more commonly leveraged collective support for resistance than individual resistance. Leveraging supports as a resistance strategy is similar to the help-seeking approach frequently reported in previous studies. For example, Hunter et al. (2004) found that students may seek help from teachers, parents, and friends in school bullying. The present study gives a deeper explanation that students were more willing to seek support from teachers than from parents, with friends being the least consulted. Research suggests teachers, as school authorities, may be perceived as reliable conflict resolvers (Aceves et al., 2010). However, some studies suggest help-seeking from parents or peers may also be perceived as useful (Healy & Sanders, 2018). Another finding of this study is that some victims hesitated to seek support from teachers due to considerations related to the bullying situation, such as considerations about bullying escalation, which may suggest that contextual factors, such as interpersonal relationships and victims’ goals in addressing bullying, may influence decisions about help-seeking (Wachs et al., 2020; Yablon, 2012). Individual resistance, including counterattacking, retaliation to alter bully behavior, or changing victims’ own appearance, has also been identified as a coping strategy in multiple studies (Marlow et al., 2023; Prince et al., 2024).

### 4.2. Victims’ Coping Strategies Integrating Developmental and Cultural Mechanisms

The findings suggest that coping strategies do not develop linearly across students’ development; rather, coping repertoires are gradually reconstructed through shifts in behavior, cognition, emotion, and socialization. Elementary school students were more likely to use immediate emotional release, fantasizing, and negative self-perceptions, and to leverage support from adults as a primary coping resource. This could be understood in light of limited cognitive appraisal abilities. Younger students exhibited helplessness, avoidance, withdrawal, wishful thinking, and low self-esteem, alongside uncertainty about resolving bullying. However, some did resist using personal strength, as evidenced in current findings (Bijttebier & Vertommen, 1998; Hunter & Boyle, 2004; Hunter et al., 2007). Even when internalizing emotions, elementary students released anger and anxiety more directly before seeking adult support. This demonstrates the external locus of control characteristic of this developmental stage (Bijttebier & Vertommen, 1998; Hunter & Borg, 2006).

During middle school, students continued to experience negative self-perceptions but showed a shift toward greater self-focus and internalized coping strategies, including attempts at self-change, bullying acquiescence, and emotional involution. At this stage, reliance on adult support decreased, and students were less likely to leverage the support of peers. This stage represents a transitional phase in the developmental trajectory of coping, where distress expression becomes more contained and cognitively mediated. Influenced by prior bullying experiences, school climate, social evaluation, identity development, and fears of escalation consequences, students tend to adopt more silent coping strategies, such as pretending not to care, doing nothing, engaging in introspection, or swallowing their emotions (Cassidy, 2008; Hunter & Borg, 2006; Mischel & Kitsantas, 2019; Smith & Caron, 2022). Coping approaches show greater regulation and relative stability compared to elementary school, yet remain fragile and context-dependent. When bullying occurs frequently or over an extended period, or when middle school students perceive it as beyond their control, they may still turn to adult support; however, some hesitate to seek social support due to fears of appearing weak, or shame, low confidence, or concerns about retaliation or a failure to resolve the situation (Donoghue et al., 2014; Hunter & Boyle, 2004; Skrzypiec et al., 2011).

As development progresses into high school, coping strategies turn toward being more cognitively engaged and complex. Students used more rationalization, acquiescence, or bullying avoidance and drew on personal strength or interpersonal relationships to resist bullying. Social pressures related to reputation, self-image, and diverse interpersonal relationships also guide high school students’ coping in covert, strategic, and indirect ways rather than overt or aggressive release, which facilitates them to derive strength particularly from friends (Hemphill et al., 2012; Olweus et al., 2019). High school students cope in a constructive way, which is associated with ego development in high school students, characterized by conformity to broader social expectations and the avoidance of social deviation as a defensive adaptation (Hemphill et al., 2012; Martin & Gillies, 2004; Merrill & Hanson, 2016). Supported by enhanced cognitive maturity, awareness of social status and interpersonal relationships, evolving social expectations, richer social experiences, strengthened self-confidence and self-identity, and a desire to demonstrate independence and self-control, high school students view coping with school bullying as a personal responsibility that requires rational and confident adjustment (Bibou-Nakou & Markos, 2013; Evans et al., 2017; Murray-Harvey et al., 2012; Olweus et al., 2019).

The selection and development of coping strategies may also be understood within their sociocultural context. In the self-reports, some students described regulating or suppressing their negative emotions to avoid exacerbating peer tensions or in hopes that the bullying would stop. These patterns resonate with observations from prior research on emotional regulation in East Asian educational settings (Ji & Nagata, 2024). Furthermore, some students reported turning to teachers for help, citing the teacher’s authoritative role in the classroom. This preference aligns with observations about the culture of respecting teachers more and encourages students to recognize teachers’ strengths and abilities in the Chinese context (Zhu, 2020). Integrating developmental and cultural factors, the Victims’ Coping Strategies Model conceptualizes Chinese students’ coping responses to school bullying. Compared with traditional dichotomous or tripartite frameworks that rely on a single categorical dimension to explain coping, this model connected the existing coping categorizations to organize coping strategies along multiple dimensions, including engagement–disengagement and cognitive–emotional versus cognitive–behavioral approaches, more comprehensively capturing the complex interplay between developmental processes and culturally embedded influences in the Chinese context (Compas et al., 2001; Davis et al., 2014; Kokkinos et al., 2014; Lazarus & Folkman, 1984; Roth & Cohen, 1986; Rudolph et al., 1995; Sontag & Graber, 2010; Weisz et al., 1995).

Grounded in authentic self-reports of Chinese students’ school bullying experiences, the Victims’ Coping Strategies Model contributes theoretically to a broader understanding of coping as a developmentally and culturally adaptive system that extends beyond the boundaries of single-orientation traditional models. One of the implications of the model is support for teachers, parents, and counselors to recognize the variability and complexity of victims’ coping strategies in Chinese school bullying. Not all incidents of school bullying require adult intervention. However, when victims seek help from teachers, timely support leverage, while considering the individual’s developmental stage, the specific school bullying context, and the coping strategies they are likely using, is essential when students need it (Bjereld et al., 2025). At the same time, these findings suggest that facilitating a positive school climate may involve both collaborative protective factors between families and schools and strong teacher–student relationships through restorative practices that emphasize the student’s voice to enhance school bullying coping effectiveness (Gregory et al., 2024; Matuschka et al., 2021). Anti-bullying programs can be designed to support students’ multiple coping strategies since school bullying dynamics change and are aligned with sociocultural and developmental contexts (Dowling & Carey, 2013). Regular mental health screening and preventive guidance can further promote adaptive and effective coping while reducing the risk of victimization involution in school bullying (Matuschka et al., 2021).

This study systematically summarized the coping strategies used by Chinese college students who experienced school bullying from elementary to high school and constructed a model of these strategies. Several limitations were acknowledged in this study, suggesting avenues for future research. To capture a holistic view of coping strategies, this study relied on the retrospective recall of Chinese college students’ school bullying experiences, which may have included inaccurate and ambiguous memories. Future research could directly sample current elementary, middle, and high school students to examine coping strategies and developmental changes from a first-person, real-time perspective. Moreover, although this study identified stage-related differences in coping strategies across development, it relied on a cross-sectional design; therefore, a longitudinal study could be adopted in the future to monitor changes in coping strategies over time. Third, incorporating broader social-ecological perspectives and contextual factors, such as school policies, anti-bullying interventions, and variations in school climate and community cultural characteristics, would further enrich the Victims’ Coping Strategies Model (Espelage, 2015).

### 4.3. Implications and Future Directions

This study constructs a coping model that integrates cultural context and developmental stages, transcending simplistic binary classifications such as active/passive responses. By integrating dimensions of “engagement-disengagement” and “cognitive-affective/behavioral”, the model forms a four-quadrant framework for victim coping strategies. The model illustrates how tensions among coping strategies can emerge, for example, cognitive reappraisal coexists with emotional reactivity or disengaged responses within a unified theoretical framework.

The model illustrates the interplay between internal psychological states and externally observable behaviors, while emphasizing how cultural and developmental factors jointly shape response patterns, which provides a more nuanced analytical lens for examining victim coping in interpersonal stress contexts, moving beyond oversimplified categorizations to account for the complexity and variability inherent in human response strategies. These findings underscore that coping with bullying is context-dependent rather than a static personal trait.

From a practical perspective, this model functions as a diagnostic and intervention framework for educators, counselors, and parents, emphasizing that effective support must align with students’ existing developmental coping strategies rather than imposing universal “correct” methods. For students prioritizing emotional response strategies, particularly younger children, a focus should be placed on socio-emotional support and safe expression environments to help transition the child from overwhelming emotional involution or release to regulated responses rather than demanding immediate behavioral change (Sani et al., 2025). When students rely on endurance and avoidance strategies, this may reflect perceived powerlessness or the lack of a sense of safety, suggesting the potential importance of supportive school polices, climates, and measures needed (Olsson et al., 2025). Anti-bullying policies could foster school climates where avoidance becomes irrational, while individual interventions build self-efficacy and incrementally empower safe boundary-testing. For victims engaging in cognitive reconstruction, particularly middle and high school students, the data indicate that guidance in positive cognitive reappraisals may help prevent negative interpretations of the school bullying, such as negative self-perception or self-blame (Vacca et al., 2023). Action-oriented resistance may be more likely to occur when help-seeking pathways are perceived as responsive and supportive (Gregory et al., 2024). It is important to increase awareness among adults, parents, and students about the value of providing constructive and empathetic responses when school bullying occurs.

This study suggests key areas for future investigation based on its limitations and findings. First, while retrospective narratives provide valuable depth, they are prone to memory distortion. Longitudinal designs that follow students from elementary through high school are essential to validate observed developmental changes in real time. Second, the four-quadrant model needs empirical testing via standardized measurement tools. Such instruments would allow researchers to assess coping patterns at scale and explore their links to mental health outcomes. Finally, adopting an expanded ecological framework (Espelage, 2015) is recommended. This includes incorporating perspectives from bullies and bystanders within the same cultural context and analyzing how school-level variables, like anti-bullying program structures or overall campus climate, affect students’ choice and effectiveness of coping strategies. Cross-cultural comparisons would help pinpoint how specifically Chinese cultural norms influence these adaptive processes. Addressing these areas will enhance both theoretical clarity and practical relevance for anti-bullying interventions.

## 5. Conclusions

This study investigated how Chinese students navigate school bullying, constructing a theoretically grounded framework from the lived experiences of 67 college students. Retrospective accounts revealed diverse coping strategies, categorized into four domains: emotional response, endurance and avoidance, cognitive reconstruction, and action-oriented resistance. Rather than portraying victims as passive in school bullying, these self-reports illustrate the different ways Chinese students manage interpersonal stress and psychological imbalance.

Grounded in the existing coping literature, this study proposes a four-quadrant Victims’ Coping Strategies model structured along engagement–disengagement and cognitive–affective/behavioral axes. It extends binary frameworks into a more integrative system, capturing the nuanced interplay between internal states and external actions, such as how students might cognitively resist while behaviorally avoiding conflict, or how they emotionally disengage as a form of self-protection.

Compared with previous coping frameworks that focus on a single educational stage and pay limited attention to cultural context, this model incorporates both developmental and cultural factors in the Chinese context. Based on retrospective accounts, victims in China reported using more direct and externally focused coping strategies in their memories of elementary school, whereas their recollections of coping in middle and high school were described as more internalized, cognitively complex, and socially strategic. These reported shifts highlight perceived patterns rather than established developmental trajectories. The coping strategies used, such as emotional expression (e.g., emotional involution) and help-seeking behaviors (e.g., reliance on teacher authority), may potentially reflect Chinese cultural contexts that emphasize harmony and respect for authority.

Finally, these findings underscore the importance of moving beyond one-size-fits-all recommendations for victims of bullying. School bullying intervention lies not in teaching a single “best” method, but in creating safe, responsive school environments where students can effectively cope with stressful situations. By categorizing and modeling the coping strategies reported by students in China, this study may help educators and school practitioners better recognize students’ coping efforts and support against school bullying.

## Figures and Tables

**Table 1:** Coding of subjective reports on school bullying status and response strategies.

Category	Node	Proportion	Coding Instructions
Bullying Type	5	11.63%	“physical bullying”, “social bullying”, “verbal bullying”, “cyberbullying”, “theft or destruction of property”
Educational Stages of Bullying Occurrence	3	6.98%	“elementary school”, “middle school”, “high school”
Location of Bullying	7	16.28%	“dormitory”, “classroom”, “toilet”, “corridor/playground”, “on the way to/from school”, “off-campus venues”, “social-media platforms”
Who is the Bully	4	9.30%	“same-grade classmates”, “older students”, “students from other schools”, “youth outside school”
Bullying Frequency	3	6.98%	“frequent bullying”, “multiple incidents”, “a single incident”
Emotional Response	2	4.65%	“emotional involution”: fear (scared, lingering fear, worried, etc.); sadness (upset, wronged, anguished, humiliated, etc.); despair; loathing “emotional release”: crying (uncontrollable tears, sobbed uncontrollably, had a good cry, often tearful, etc.); venting (anger, irritability, shouting, etc.)
Cognitive Reconstruction	2	4.65%	“rationalization of bullying” (making excuses for bullies, rationalizing bullying behavior) “negative self-perception” (self-deprecation, self-efficacy decreased, lower self-esteem, and self-worth)
Action-Oriented Resistance	2	4.65%	“individual resistance” (resistance to change others, resistance through self-change) “leveraging support for resistance” (leveraging support from teachers, leveraging support from parents, leveraging support from friends)
Endurance and Avoidance	3	6.98%	“bullying acquiescence” (passive compliance, compliant silence, silent endurance, appeasement); “bullying avoidance” (physical distancing from bullies, trying to forget the bullying); “bullying fantasizing” (imagining the bullying never happened, wishing victim traits would vanish, fantasizing the bullies were punished)
The Bullying Outcome	4	9.30%	“persistent bullying”, “escalated bullying”, “decreased bullying”, “cessation of bullying”
Causes of Bullying Stopping	3	6.98%	“bullies became afraid”, “bullies showed approval”, “victim submission”
Causes of Bullying to Continue	5	11.63%	“ineffective help-seeking” (teacher/school neglect, ineffective punishment of bullies); “insufficient policy diffusion of school anti-bullying rules” (inconsistent teacher attention, unclear or uneven anti-bullying policy); “bystander effect” (bystander inaction or bystander reinforcement); “bully’s empathetic failure” (moral disengagement, bully’s peer influence); “lack of coordinated safeguard” (no home–school or intra-school joint action).

**Table 2:** Domains, categories, frequency, and ranking of school bullying coping strategies.

Domains	Categories	Reported Times	Ranking
Emotional Response Strategy (66 segments)	Emotional Release	18 segments	3
	Emotional Involution	48 segments	1
Endurance and Avoidance Strategy (37 segments)	Bullying Acquiescence	15 segments	5
	Bullying Avoidance	10 segments	7
	Bullying Fantasizing	12 segments	9
Cognitive Reconstruction Strategy (29 segments)	Rationalization for Bullying	13 segments	8
	Negative Self-Perception	16 segments	4
Action-Oriented Resistance Strategy (47 segments)	Individual Resistance	14 segments	6
	Resistance to Change Others	7 segments	
	Resistance through Self-change	7 segments	
	Leveraging Support for Resistance	33 segments	2
	Leveraging Support from Teachers	15 segments	
	Leveraging Support from Parents	12 segments	
	Leveraging Support from Friends	6 segments	

Note. Frequencies represent coded narrative segments instead of individual participants.

**Table 3:** School bullying coping strategies distribution by developmental stages.

Coping Strategies	Reported Times		
	Elementary School	Middle School	High School
Emotional Response Strategy	26/66 (39.4%)	22/66 (33.3%)	18/66 (27.3%)
Emotional Release	12/18 (66.7%)	4/18 (22.2%)	2/18 (11.1%)
Emotional Involution	14/48 (29.2%)	18/48 (37.5%)	16/48 (33.3%)
Endurance and Avoidance Strategy	16/37 (43.2%)	10/37 (27.0%)	11/37 (29.7%)
Bullying Acquiescence	2/15 (13.3%)	6/15 (40.0%)	7/15 (46.7%)
Bullying Avoidance	3/10 (30.0%)	3/10 (30.0%)	4/10 (40.0%)
Bullying Fantasizing	11/12 (91.7%)	1/12 (8.3%)	0/12 (0%)
Cognitive Reconstruction Strategy	9/29 (31.0%)	12/29 (41.4%)	8/29 (27.6%)
Rationalization for Bullying	2/13 (15.4%)	5/13 (38.5%)	6/13 (46.2%)
Negative Self-Perception	7/16 (43.8%)	7/16 (43.8%)	2/16 (12.5%)
Action-Oriented Resistance Strategy	16/47 (34.0%)	13/47 (27.7%)	18/47 (38.3%)
Individual Resistance	3/14 (21.4%)	5/14 (35.7%)	6/14 (42.9%)
Resistance to Changing Others	2/7 (28.6%)	2/7 (28.6%)	3/7 (42.9%)
Resistance through Self-change	1/7 (14.3%)	3/7 (42.9%)	3/7 (42.9%)
Leveraging Support for Resistance	13/33 (39.4%)	8/33 (24.2%)	12/33 (36.4%)
Leveraging Support from Teachers	6/15 (40.0%)	5/15 (33.3%)	4/15 (26.7%)
Leveraging Support from Parents	5/12 (41.7%)	4/12 (33.3%)	3/12 (25.0%)
Leveraging Support from Friends	2/6 (33.3%)	0/6 (0%)	4/6 (66.7%)

Note. Frequencies represent coded narrative segments instead of individual participants.

## Data Availability

The raw data supporting the conclusions of this article will be made available by the authors on request.
